# Comparison of nanoparticle-mediated transfection methods for DNA expression plasmids: efficiency and cytotoxicity

**DOI:** 10.1186/1477-3155-9-47

**Published:** 2011-10-20

**Authors:** María Carolina Durán, Saskia Willenbrock, Annette Barchanski, Jessika-M V Müller, Arianna Maiolini, Jan T Soller, Stephan Barcikowski, Ingo Nolte, Karsten Feige, Hugo Murua Escobar

**Affiliations:** 1 Equine Clinic, University of Veterinary Medicine Hannover, Buenteweg 9, 30559 Hannover, Germany; 2 Small Animal Clinic and Research Cluster of Excellence "REBIRTH", University of Veterinary Medicine, Buenteweg 9, 30559 Hannover, Germany; 3 Laser Zentrum Hannover e.V., Hollerithallee 8, 30419 Hannover, Germany; 4 Institute of Veterinary Medicine, University of Goettingen, Burckhardtweg 2, 37077 Goettingen, Germany; 5 Institute of Technical Chemistry I, University of Duisburg-Essen and Center for Nanointegration Duisburg-Essen (CeNIDE), Universitaetsstr. 5-7, 45141 Essen, Germany

## Abstract

**Background:**

Reproducibly high transfection rates with low methodology-induced cytotoxic side effects are essential to attain the required effect on targeted cells when exogenous DNA is transfected. Different approaches and modifications such as the use of nanoparticles (NPs) are being evaluated to increase transfection efficiencies. Several studies have focused on the attained transfection efficiency after NP-mediated approaches. However, data comparing toxicity of these novel approaches with conventional methods is still rare.

Transfection efficiency and methodology-induced cytotoxicity were analysed after transfection with different NP-mediated and conventional approaches. Two eukaryotic DNA-expression-plasmids were used to transfect the mammalian cell line MTH53A applying six different transfection protocols: conventional transfection reagent (FuGENE HD, FHD), FHD in combination with two different sizes of stabilizer-free laser-generated AuNPs (PLAL-AuNPs_S1,_S2), FHD and commercially available AuNPs (Plano-AuNP), and two magnetic transfection protocols. 24 h post transfection efficiency of each protocol was analysed using fluorescence microscopy and GFP-based flow cytometry. Toxicity was assessed measuring cell proliferation and percentage of propidium iodide (PI%) positive cells. Expression of the respective recombinant proteins was evaluated by immunofluorescence.

**Results:**

The addition of AuNPs to the transfection protocols significantly increased transfection efficiency in the pIRES-hrGFPII-*eIL-12 *transfections (FHD: 16%; AuNPs mean: 28%), whereas the magnet-assisted protocols did not increase efficiency. Ligand-free PLAL-AuNPs had no significant cytotoxic effect, while the ligand-stabilized Plano-AuNPs induced a significant increase in the PI% and lower cell proliferation. For pIRES-hrGFPII-*rHMGB1 *transfections significantly higher transfection efficiency was observed with PLAL-AuNPs (FHD: 31%; PLAL-AuNPs_S1: 46%; PLAL-AuNPs_S2: 50%), while the magnet-assisted transfection led to significantly lower efficiencies than the FHD protocol. With PLAL-AuNPs_S1 and _S2 the PI% was significantly higher, yet no consistent effect of these NPs on cell proliferation was observed. The magnet-assisted protocols were least effective, but did result in the lowest cytotoxic effect.

**Conclusions:**

This study demonstrated that transfection efficiency of DNA-expression-plasmids was significantly improved by the addition of AuNPs. In some combinations the respective cytotoxicity was increased depending on the type of the applied AuNPs and the transfected DNA construct. Consequently, our results indicate that for routine use of these AuNPs the specific nanoparticle formulation and DNA construct combination has to be considered.

## Background

Transfection of eukaryotic cells is a key technology in cell biology being used in several areas of basic and therapeutic research. The critical points in these experimental approaches are the achieved transfection efficiencies and the reproducibility of the performed experiments. Therefore, a stable high transfection rate with low methodology induced side effects in terms of toxicity would be desirable. Furthermore, the methods used should not interfere with the functionality of the delivered molecules such as large DNA expression plasmids or small RNAs such as siRNAs and miRNAs.

Currently, several non-viral transfection methods for eukaryotic cells are used to introduce membrane impermeable molecules into the cells. However, the efficiency, toxicity, and reproducibility, which may vary depending on the characteristics of the cells used, remain a crucial aspect in cell transfection. Consequently, various methods and modifications are currently being evaluated to increase efficiency and reduce toxicity. Thus, both novel laser-based transfection methods [1] as well as nanoparticle (NP) approaches have been evaluated in recent studies [2-4]. Considering the latter, gold Nanoparticles (AuNPs) are in the focus of intense research due to their chemical stability, electro-density and -affinity to biomolecules such as DNA, when these AuNPs are charged [5]. However, the inherent characteristics of the applied NPs could induce different toxic effects on cells due to several factors such as particle number and size, surface dose, surface coatings, degree of agglomeration, surface charges on particles and method of particle synthesis as well as post-synthetic modifications. During or after most forms of NP synthesis, the generated NPs are modified to prevent aggregation or induce disaggregation. The surface modification and surface charge can have a major impact on the biological response to various particles, therefore, the particle specific surface modification and the agents are an important factor that must be considered when choosing particular NPs [6].

The valuable characteristics of AuNPs make them suitable to act as plasmid DNA carriers and transfection enhancers. Similarly, magnetic NPs loaded with the nucleic acid of interest are used to increase transfection efficiency by applying magnetic force to the DNA-NP complexes. These magnetic DNA-NP complexes are drawn towards the outer cell membrane via magnetic force and are subsequently taken up by the cell via endocytosis.

AuNPs can be generated using various methods, most of which rely on chemical reactions or gas pyrolysis, which carry the risk of agglomeration or contamination with impurities such as citrate and residual precursors like chloroauric acid [7].

Pulsed laser ablation in liquids (PLAL) has been reported to present advantages in NP generation such as low restriction for the choice of the source material allowing the generation of highly pure colloidal particles [8]. The generated pure AuNPs with the oxidation states Au^+ ^and Au^+3 ^were reported to have a unique surface chemistry and to be free of stabilizers, as a result of the chemical composition of the liquid media used during synthesis [8]. This inherent charge given to these AuNPs, without adding a special coating that could have a potential cytotoxic effect make these NPs interesting for DNA-binding and cell transfection. Previous studies demonstrated that unmodified, circular, negatively charged DNA molecules adsorb easily onto these positively charged NPs [2]. Moreover, the incubation of these AuNPs with plasmid DNA did not alter the uptake of the vector through the plasma membrane in presence of a transfection reagent, and showed no apparent effect on the biological activity of the produced recombinant protein [9]. However, although AuNP approaches have gained popularity, the data concerning the toxic potential of these particles is still marginal and the characterisation of the toxic potential of AuNPs in combination with complex DNA expression plasmids is mostly limited to model molecules.

Herein, we analysed the transfection efficiency and cytotoxicity of different NP-mediated transfection approaches after the transfection of a mammalian cell line with two different eukaryotic expression vectors encoding simultaneously for an expression protein (canine HMGB1 or equine IL-12) and the humanized renilla Green Fluorescent Protein (hrGFP). Results were compared to those obtained using a conventional standard transfection protocol (FuGENE HD, Roche, Mannheim, Germany).

## Results

### Transfection Efficiency

#### Fluorescence Microscopy

The uptake of plasmid DNA was primarily evaluated by comparing the GFP positive cells to the total quantity of cells showing blue DAPI fluorescence dye staining, thus attaining an estimate of the transfection efficiency. After 24 h incubation, the transfection process both with the plasmid DNA and with the transfection reagents alone did not induce major negative effects on the cells. An exception to this was the addition of the Plano-AuNP to the cells, where 24 h post-transfectional cells showed advanced apoptotic signs. The transfection efficiency of cells transfected with the Plano-AuNP, PLAL-AuNP Size 1 and Size 2 protocol was apparently higher than that achieved with the conventional FHD transfection reagent or with the magnetic transfection protocols (MATra-A, MA Lipofection) (Images not shown).

#### Flow cytometry analysis of GFP expression

**Figure 1:**
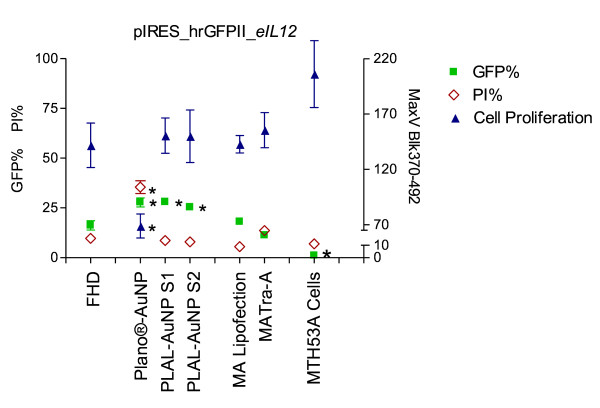
**Transfection efficiency and toxicity of pIRES-hrGFPII-*eIL-12***. GFP- (■) and PI- (◊) positive cells 24 h after transfection with pIRES-hrGFPII-*eIL-12*. Mean cell proliferation (▲) (48 h and 72 h after transfection with pIRES-hrGFPII-*eIL-12*). Each bar represents a mean ± SD. * p ≤ 0.05.

**Figure 2:**
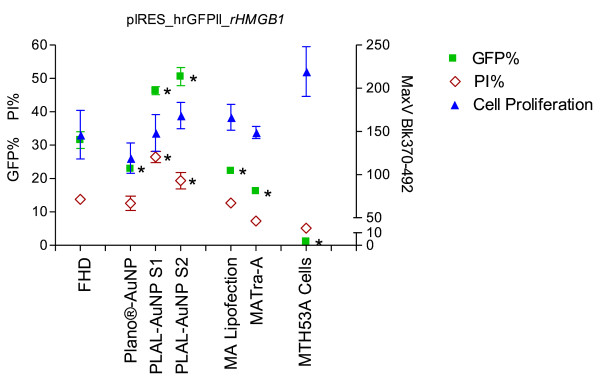
**Transfection efficiency and toxicity of pIRES-hrGFP-r*HMGB1***. GFP- (■) and PI- (◊) positive cells 24 h after transfection with pIRES-hrGFP-r*HMGB1*. Mean cell proliferation (▲) (48 h and 72 h after transfection with pIRES-hrGFP-r*HMGB1*). Each bar represents a mean ± SD. * p ≤ 0.05.

The mean transfection efficiencies of the FHD transfection were 16.22% and 31.52% for pIRES-hrGFPII-*eIL-12 *(Figure 1; Table 1) and pIRES-hrGFPII-*rHMGB1 *(Figure 2; Table 1), respectively.

**Table 1:** Transfection efficiency

	pIRES-hrGFPII-*eIL-12*	pIRES-hrGFPII-*rHMGB1*
	
	GFP %	GFP %
**FHD**	16.22 ± 9.69	31.52 ± 4.33
**Plano^®^-AuNP**	27.80 ± 3.90 *	22.93 ± 0.98 *
**LAG-AuNP S1**	28.01 ± 1.97 *	46.33 ± 2.07 *
**LAG-AuNP S2**	25.41 ± 2.22 *	50.56 ± 4.71 *
**MA Lipofection**	18.11 ± 0.60	22.29 ± 1.36 *
**MATra-A**	11.33 ± 1.30	16.24 ± 1.25 *
**MTH53A Cells**	1.98 ± 0.17	1.15 ± 0.56 *

GFP positive cells 24 h after transfection with pIRES-hrGFPII-*eIL-12 *or pIRES-hrGFP-*HMGB1*. Results are expressed as mean ± SD. * p ≤ 0.05.

When AuNPs (Plano-AuNP and PLAL-AuNPs Size 1 and 2) were added, transfection efficiencies were significantly increased for the pIRES-hrGFPII-*eIL-12 *vector, reaching an almost two fold increase with PLAL-AuNPs Size 2 and Plano-AuNP (FHD: 16.22%; PLAL-AuNPs Size 2: 27.80%; Plano-AuNP: 28.01%; Figure 1; Table 1). For the pIRES-hrGFPII-*rHMGB1 *vector a slighter but still significant increase was observed when PLAL-AuNPs Size 1 and 2 were applied (FHD: 31.52%, PLAL-AuNPs_S1: 46.33%, PLAL-AuNPs_S2: 50.56%; Figure 2; Table 1).

### Toxicity Analyses

#### Flow cytometry analysis with propidium iodide staining

For the pIRES-hrGFPII-*eIL-12 *vector the mean propidium iodide percentages (PI%) of each protocol were similar to those reached by the cells transfected with the conventional FHD protocol. An exception was the Plano-AuNP protocol, showing a three-fold increase of the mean PI% to 35.43% when compared to the FHD protocol (9.69%; Figure 1; Table 2).

**Table 2:** Transfection toxicity

	pIRES-hrGFPII-*eIL-12*	pIRES-hrGFPII-*rHMGB1*
	
	PI %	PI %
**FHD**	9.69 ± 2.92	13.75 ± 1.35
**Plano^®^-AuNP**	35.43 ± 5.53 *	12.56 ± 3.72
**LAG-AuNP S1**	8.65 ± 1.24	26.45 ± 2.93 *
**LAG-AuNP S2**	7.92 ± 0.49	19.37 ± 4.28 *
**MA Lipofection**	5.56 ± 1.43	12.67 ± 1.33
**MATra-A**	13.6 ± 3.74	7.25 ± 0.29
**MTH53A Cells**	1.14 ± 0.17	1.01 ± 0.28

PI positive cells 24 h after transfection with pIRES-hrGFPII-*eIL-12 *or pIRES-hrGFP-*HMGB1*. Results are expressed as mean ± SD. * p ≤ 0.05.

Transfection of the pIRES-hrGFPII-*rHMGB1 *vector with the different protocols resulted in significantly higher PI% using the PLAL-AuNPs_S1 and _S2. The PLAL-AuNPs_S1 (PI 26.45%) showed a PI% nearly twice that of the FHD protocol (13.75%; Figure 2; Table 2).

#### Proliferation Assay

The effect of the different transfection protocols on cell vitality was investigated by determining cell proliferative activity with a standard proliferation test (Cell Proliferation ELISA BrdU (colorimetric), Roche Diagnostics, Mannheim, Germany). The BrdU incorporation assayed 48 h after transfection was significantly reduced when pIRES-hrGFPII-*eIL-12 *was transfected using the Plano-AuNP and the PLAL-AuNPs_S2 protocol. Seventy-two hours after transfection, a decreased BrdU incorporation was observed in the Plano-AuNP and in the FHD transfection protocols (Figure 1; Table 3). The pIRES-hrGFPII-*rHMGB1 *transfections showed a significantly reduction in incorporation of BrdU 48 h after transfection using the PLAL-AuNPs_S1 protocol. Similar results were observed for the FHD and Plano-AuNP protocols 72 h post transfection (Figure 2; Table 3).

**Table 3:** Cell proliferation after transfection

	pIRES-hrGFPII-*eIL-12*		pIRES-hrGFPII-*rHMGB1*	
	**48 h**	**72 h**	**48 h**	**72 h**

**FHD**	172.57 ± 53.44	111.06 ± 18.72*	201.13 ± 52.57	91.23 ± 1.04*
**Plano^®^-AuNP**	72.99 ± 39.32*	64.65 ± 14.19*	154.49 ± 28.71	83.81 ± 8.34*
**LAG-AuNP S1**	126.19 ± 41.31	174.86 ± 18.54	103.00 ± 21.84*	193.48 ± 14.05
**LAG-AuNP S2**	98.95 ± 25.09*	200.93 ± 7.52	140.53 ± 30.20	196.35 ± 15.79
**MALipofection**	132.24 ± 21.05	153.30 ± 12.38	153.17 ± 47.41	179.62 ± 24.20
**MATra-A**	165.15 ± 42.89	145.88 ± 40.31	143.72 ± 22.50	153.77 ± 13.07
**MTH53A Cells**	191.84 ± 25.75	188.01 ± 20.11	185.07 ± 21.15	178.11 ± 21.01

Cell proliferation 48 h and 72 h after transfection with pIRES-hrGFPII-*eIL-12 *or pIRES-hrGFP-*HMGB1*. Results are expressed as mean absorbance values ± SD. * p ≤ 0.05.

### Protein Expression

#### Protein expression detection via immunofluorescence

**Figure 3:**
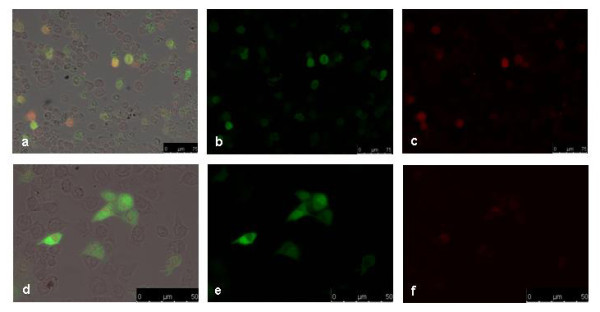
**Immunofluorescence 24 h after transfection**. pIRES-hrGFPII-*eIL-12 *transfection with the FHD protocol, primary antibody goat IgG anti-p35 and a donkey anti-goat secondary antibody (Texas Red fluorochrome). (a) GFP and Red Fluorescence merged image, (b) GFP Fluorescence and (c) Red Fluorescence images. Scale bar 50 μm. pIRES-hrGFP-HMGB1 transfection with the FHD protocol, primary antibody mouse anti-HMGB1 and secondary antibody goat anti-mouse (Texas Red fluorochrome). (d) GFP and Red Fluorescence merged image, (e) GFP Fluorescence and (f) Red Fluorescence images. Scale bar 75 μm.

Control cells showed only background staining, whereas cells transfected with pIRES-hrGFPII-*eIL-12 *revealed a diffuse accumulation of eIL-12 protein in the cytoplasm and nuclei (Figure 3a-c). Cells transfected with pIRES-hrGFPII-*rHMGB1 *showed a concentration of HMGB1 protein located in the nuclei (Figure 3d-f). Transfection of the cells with the pIRES-hrGFPII-*eIL-12 *or the pIRES-hrGFPII-*rHMGB1 *vector led to the expression of biological functional recombinant proteins localized in their final destination.

**Figure 4:**
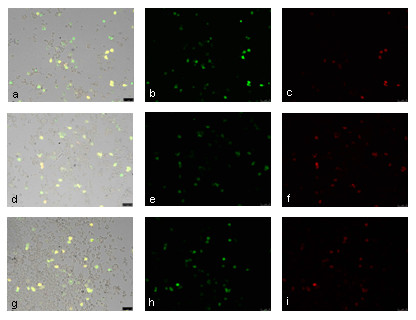
**Immunofluorescence 24 h after NP-mediated transfection**. pIRES-hrGFP-HMGB1 transfection with NP-mediated protocols. Plano-AuNP (a, b, c), PLAL-AuNP Size 2 (d, e, f), and MA Lipofection (g, h, i)). Primary antibody: mouse anti-HMGB; secondary antibody: goat anti-mouse (Texas Red fluorochrome). a, d, g: GFP and Red Fluorescence merged image; b, e, h: GFP Fluorescence and (c, f, i) Red Fluorescence images. Scale bar 75 μm.

The transfections using both gold NP and Ma Lipofection protocols in combination with pIRES-hrGFPII-*rHMGB1 *showed a HMGB1 protein expression similar to the FHD protocol (Figure 4).

## Discussion

Advances in immunology and cancer research would benefit from improved transfection efficiencies, high reproducibility and low toxicity of the required transfection approach. High transfection efficiency for plasmid DNA delivery into cells is still an important issue in gene therapy. Thus, a number of different approaches have been used to increase efficiency [10-12]. Unfortunately, the majority of the studies involving transfection of mammalian cells with non-viral vectors primarily assess transfection efficiency, lacking toxicity data. Therefore, the present study compared several NP-mediated transfection protocols in which plasmid DNA vectors were transfected into a mammalian cell line and the transfection efficiency and cytotoxicity of each protocol was analysed after transfection.

The addition of AuNPs (PLAL-AuNPs_S1 and _S2 and Plano-AuNPs) to the pIRES-hrGFPII-*eIL-12 *transfection protocols significantly increased transfection efficiency (FHD: 16%; AuNP transfection efficiency mean: 28%; p = 0.05). Compared to this, the magnet-assisted protocols did not improve the transfection efficiency of pIRES-hrGFPII-*eIL-12*, resulting in values similar to the FHD protocol. An increase of the transfection efficiency for the pIRES-hrGFPII-*rHMGB1 *was only detectable with the PLAL-AuNPs (FHD: 31%; PLAL-AuNPs_S1: 46%; PLAL-AuNPs_S2: 50%; p = 0.05). As for pIRES-hrGFPII-*eIL-12*, with the recombinant pIRES-hrGFPII-*rHMGB1 *vector no improvement of transfection efficiency was achieved through the use of the magnet-assisted transfection protocols. On the contrary, the efficiency was significantly lower when compared to the conventional FHD protocol.

Remarkably, the AuNP-mediated transfection efficiencies achieved in this study are higher than those reported by Schakowski *et al*. (2001) [12] in which a colon carcinoma cell line was transfected with minimal size gene transfer (MIDGE) vectors and corresponding plasmids (containing coding sequences for *eGFP *or human *IL-2*). Here, the transfection efficiency was up to 36% (MIDGE Vectors) and 33% (plasmid vectors) respectively [12]. A previous study by Petersen *et al*. (2009) [2] reported an apparent increase of the transfection rates when the biocompatibility of PLAL-AuNPs was analysed. The transfection reactions with plasmid DNA and PLAL-AuNPs of different hydrodynamic size classes (14, 24, 59 and 89 nm) showed transfection efficiencies ranging from 10 to 60%, reaching the highest efficiency using a NP size of 59 nm [2]. With regard to the many potential applications of these PLAL-AuNPs in the fields of research and therapy, the promising results described above indicated the necessity of analysing the definitive transfection efficiencies and the possible cytotoxicity of PLAL-AuNPs. Two of the former four PLAL-AuNPs size classes were selected for our experiments based on the results of Petersen *et al*. [2]. The chosen AuNP sizes should be considered relevant to the transfection outcome. The results of Chithrani *et al*. [13] showed that for mammalian cells (HeLa) the maximum uptake of spherical and rod-shaped AuNPs, in a size range of 10-100 nm (fully or partially modified by citric acid ligands), was reached with the 50 nm AuNPs (Feret diameter).

The transfection efficiencies for both expression vector constructs used in our study were similarly affected by the different protocols applied. The overall higher transfection efficiencies attained using the pIRES-hrGFPII-*rHMGB1 *vector could be explained due to the different vector and insert sizes. The pIRES-hrGFPII-*rHMGB1 *vector has a size of 5531 bp whereas pIRES-hrGFPII-*eIL-12 *has a molecular length of 7709 bp. Such size mediated effects in transfections were studied by Yin *et al*. (2005) [14]. They demonstrated an inverse correlation between the construct size and the promoter/enhancer activity measured by the dual luciferase system in a transient transfection assay of mammalian cells. Larger plasmid or recombinant plasmid constructs resulted in lower transfection efficiencies than when smaller ones were used [14].

In the present study, in contrast to our expectation, the magnet-assisted protocols using magnetic nanoparticle-mediated DNA-uptake did not increase the transfection ratio of pIRES-hrGFPII-*eIL-12*, resulting in transfection efficiencies and PI% comparable to those achieved by the FHD protocol. When pIRES-hrGFPII-*rHMGB1 *was transfected, the efficiency was significantly lower than that reached with the conventional FHD protocol, but with significantly lower toxicity results. A study by Bertram [3] suggested that the directed delivery of the cargo (e.g. DNA) towards the cells applying magnet-assisted transfection technology may increase the overall transfection efficiency depending on the cell type used. Although an improvement of the transfection efficiency could not be observed using the magnet-assisted protocol, it is important to highlight that as published by Renker *et al*. [15], in our study, when pIRES-hrGFPII-r*HMGB1 *was transfected using the MATra-A transfection protocol, a significantly low PI% and a cell proliferation similar to non-transfected control cells was detected. This attribute of the MATra-A protocol should be taken into consideration when gentler transfection methods on sensitive cells are required.

The protein expression results for canine HMGB1 and eIL-12 show that the protein expression is sufficient. After transfection, the expression of simple proteins as GFP and the nuclear acting HMGB1 and of complex proteins consisting of two separate subunits as IL-12 is possible. Furthermore, the addition of NP or magnetic reagent to the pIRES-hrGFPII-r*HMGB1 *transfections did not interfere with protein expression as shown in Figure 4.

Even though the use AuNPs improved the transfection efficiency achieved in this study, the required amount of reagent and type of enhancers (e.g. AuNPs) must be considered specifically for each cell type and vector in order to achieve an appropriate recombinant vector expression without incurring cell toxicity. Despite the potential benefits of the AuNPs described, the safety of their use in biological organisms has to be evaluated in full. In this study, when the pIRES-hrGFPII-*eIL-12 *vector was transfected, the addition of the ligand-free PLAL-AuNPs (S1 and S2) had no significant toxic effect on the cells. Nevertheless, when commercially purchased poly-L-lysine-coated colloidal gold NPs (Plano-AuNP) were applied, an increased PI% and decreased cell proliferation could be observed confirming a toxic effect of these particle formulations on cell vitality. For the pIRES-hrGFPII-*rHMGB1 *transfections a significantly higher PI% was measured when PLAL-AuNPs (S1 and S2) were applied. This was not supported by the cell proliferation analysis where a NP-mediated toxic effect was observed neither 48 h nor 72 h after transfection.

The potential toxicity of AuNPs has been an issue in previous studies [4,16-18]. Recently, the uptake of ligand-free positively charged gold NPs during coincubation with a bovine cell line (GM7373) occurred apparently by diffusion [19]. At the same time, the assessment of cell morphology, membrane integrity, and apoptosis revealed no AuNP-related loss of cell vitality at gold concentrations of 25 μM or below, and no cytotoxic effect was observed in a proliferation assay after exposing low cell numbers to the same PLAL-AuNP concentrations [19]. Interestingly, cell proliferation was reduced when cells were coincubated with ligand-free gold NPs concentrations of 50 μM and above [19]. Although, AuNP cytotoxicity was not the aim of the study by Petersen *et al*. [2], they observed that the PLAL-AuNP application apparently had no cytotoxic effect, since normal cell density and appearance in all set ups was similar prior- and posttransfectional. In this context, Shukla *et al*. (2005) [20] concluded that chemically synthesized AuNPs (35 ± 7 Å in size, Feret diameter) are inert and nontoxic to the cells and that no stress-induced secretion of proinflammatory cytokines as TNF-α and IL-1β by macrophage cells (RAW264.7) was detectable.

In our study, the average PI% of the transfected cells (12.3% for pIRES-hrGFPII-eIL-12; 13.9% for pIRES-hrGFPII-*rHMGB1*) can be compared with the 10-20% reported by Schakowski *et al*. [12] after the transfection of a colon carcinoma cell line with plasmid and MIDGE vectors. Regarding the size of NPs in relation to cell toxicity, Pernodet *et al*. (2006) [21] demonstrated that 13 nm AuNPs (Feret diameter) generate apoptosis and morphological deformation at 2-6 days in CF-31 human dermal fibroblast cells. Additionally, Pan *et al*. (2007) [16] reported that AuNPs with a diameter of 2 nm or less (Feret diameter) were cytotoxic for different cell lines (termed HeLa, SK-Mel28, L929 mouse fibroblasts and J774A1 mouse monocytic/macrophage cells), whereas 15 nm AuNPs were nontoxic to the cells. These NP size dependent results could be due to the larger surface area per unit mass of smaller sized NPs. Related to this, particle toxicology suggests that, for toxic particles generally, more particle surface equals more toxicity [6].

Interestingly, the significant toxicity we observed when using the 20 nm Plano-AuNP (with pIRES-hrGFPII-*eIL-12*) differs from the recent study by Brandenberger *et al*. [22]. They applied similar commercially available aqueous colloidal AuNPs, 15 nm in size and coated with poly-L-lysine. The AuNPs entered the cells, but no cytotoxic effects of these AuNPs were observed [22]. These results suggest that possibly the poly-L-lysine coating does not induce a direct toxic effect on cells, although impurities in the AuNP colloid formulations are supposed to increase the toxicity compared to pure AuNPs.

**Figure 5:**
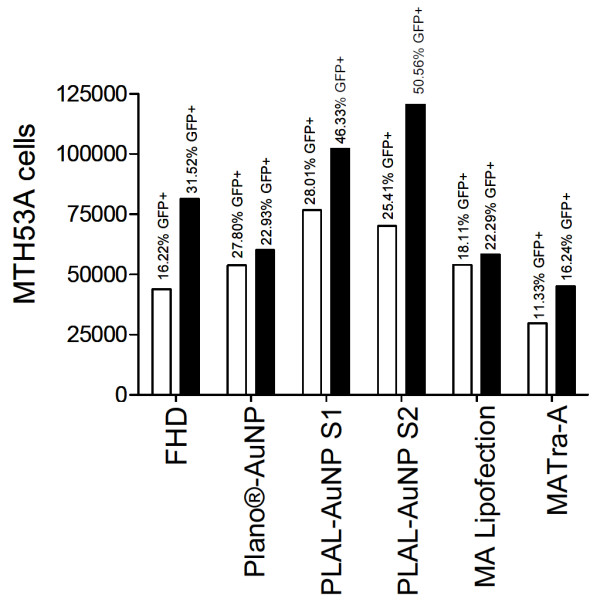
**Vital cells after transfection**. Number of vital MTH53A cells (GFP positive cells minus PI positive cells) 24 h after transfection with pIRES-hrGFP-eIL12 (□) or pIRES-hrGFP-rHMGB1 (■).

The results presented herein suggest that further use of each protocol should be evaluated under consideration of the transfection efficiency results together with the toxicity results. To do so, we subtracted the PI% from the total number of GFP positive cells (Figure 5). For the pIRES-hrGFPII-*eIL-12 *transfections, this calculation showed that even though the Plano protocol generated almost the highest transfection efficiency, the outcome was not as good when considered in combination with the cell toxicity results. In contrast, the PLAL-AuNP_S1 protocol provided the best overall (combined) results. For the pIRES-hrGFPII-*rHMGB1 *transfections the use of the PLAL-AuNPs_S2 protocol showed the highest efficiency and just a slightly increased toxicity, making this protocol the one with the best final outcome.

Hence, both test series (Figure 1 and 2, Table 1) indicate that AuNPs, in particular the physically made pure colloids, are able to significantly increase transfection efficiency and that a trade-off in cell vitality becomes significant in particular with the chemically made AuNPs. The residual nanoparticle ligands of these NPs may play an unintended, yet underestimated role in NP-mediated cellular uptake. However, further studies with different cell lines and expression vectors should be performed to be able to decide if the observed cytotoxic effects can be explained by simple NP cell intolerance or by incompatibility of the cells with the transfected recombinant vector or the expressed recombinant protein.

## Conclusions

Transfection efficiency of plasmid DNA vectors can be significantly improved by the addition of ligand-free PLAL-AuNPs (29 nm and 52 nm in size) to conventional transfection reagents like FuGENE HD. Cell vitality was negatively affected mainly by the addition of chemically generated AuNPs (Plano-AuNPs), but also slightly by physically made AuNPs (PLAL-AuNPs_S1) resulting in increased cytotoxic effects and reduction of cell proliferation. Among the transfection methods investigated comparatively in this study, 29 nm AuNPs made by PLAL span the widest window in terms of high transfection efficiency with minimized trade-off in vitality.

## Methods

### Mammalian expression vectors

Two different mammalian expression vectors simultaneously encoding for an expression protein (canine HMGB1 (HMGB1) or equine IL-12 (eIL-12)) and the hrGFP were constructed. The expression of the inserted genes of interest can be assessed by the simultaneous but separate expression of hrGFP due to a bicistronic expression cassette in the respective pIRES-*hrGFPII *plasmids used here. Accordingly, the successful transfection of the cells may be analysed using GFP-based fluorescence microscopy as well as flow cytometry. The used vectors differ in that, apart from the GFP, the HMGB1 vector encodes a single chain protein, while the IL-12 vector encodes a complex protein consisting of two different subunits which are posttranslationally processed by the cell to a joint complex. Thus, a successful assembling of recombinant IL-12 is dependent on the ability of the transfected cell to correctly process complex posttranslational protein modifications.

#### PIRES-hrGFPII-*eIL-12*

DNA encoding for eIL-12 (Vetsuisse-Faculty, University of Zurich) was amplified by PCR (primer pair: NotI_IL-12_f 5'-CGGCGGCCGCATATGTGCCCGCCGCGC-3' (forward primer); NotI_IL-12_r 5'-CGGCGGCCGCAACTGCAGGATACGG-3' (reverse primer)). The DNA contains the p35 and p40 IL-12 subunit cDNAs (p35: Acc. No. Y11129; p40: Acc. No. Y11130) separated by an IRES element, both IL-12 subunits are translated separately and then processed by the cell to a joint complex. The PCR products were separated on a 1.5% agarose gel, eluted using QIAquick Gel Extraction Kit (QIAGEN, Hilden, Germany), and cloned into the bicistronic pIRES-hrGFPII mammalian expression vector (Stratagene, La Jolla, CA, USA). Verification of the constructed plasmid was done by NotI restriction digest and sequencing.

#### PIRES-hrGFPII-*rHMGB1*

For construction of the pIRES-hrGFPII-*rHMGB1 *expression plasmid, the canine *HMGB1 *coding sequence (Acc. No. AY135519) without the terminal stop codon was inserted into the bicistronic pIRES-hrGFP II vector (Stratagene, La Jolla, CA, USA). Expression of the inserted HMGB1 coding sequence results in an HMGB1 fusion protein with a recombinant short 3 × FLAG peptide sequence at its C-terminal part (*rHMGB1*).

The following primer pair was used for PCR-amplification: EcoRI-B1-CFA-Fwd (5'-GGAATTCCACCATGGGCAAAGGAGA-3'; forward primer) and NotI-B1-CFA-Rev/-TAA (5'-AAGAATGATGATGATGAAGCGGCCGCGC-3', reverse primer).

The amplified PCR product was separated on a 1.5% agarose gel, purified using QIAquick Gel Extraction Kit (QIAGEN, Hilden, Germany) and ligated into the pIRES-hrGFPII vector plasmid (Stratagene, La Jolla, CA). Verification of the constructed plasmid was done by NotI/EcoRI double restriction digest and sequencing.

### Cell culture and in vitro transfection assays

The MTH53A canine mammary cell line used for the experiments was derived from epithelial healthy canine mammary tissue.

Eight hours prior to the transfection, 3 × 10^5 ^MTH53A cells were seeded in 6-well plates with 2 ml cell culture medium. The cells were grown as adherent cultures in a humidified atmosphere at 37°C and 5% CO_2 _in complete medium 199 (medium 199; Invitrogen, Karlsruhe, Germany) supplemented with 10% heat-inactivated fetal calf serum (PAA Laboratories GmbH, Pasching, Austria), 200 U/ml penicillin and 200 ng/ml streptomycin (Biochrom AG, Berlin, Germany)).

#### For transfection the following different protocols were applied in triplicate

**Figure 6:**
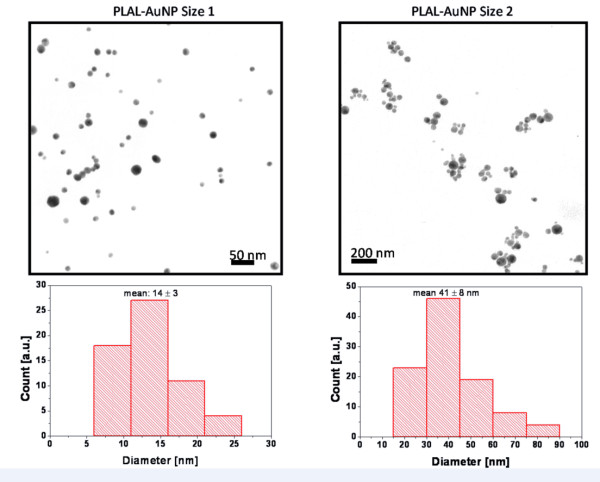
**Size distribution of pulsed laser ablation in liquid generated AuNPs**. Size distribution (Feret diameter) of PLAL-AuNP size 1 (hydrodynamic sizes: d50 = 28.5 nm and d90 = 43.4 nm, Feret diameter: 14 ± 3 nm) and size 2 (hydrodynamic sizes: d50 = 52.4 nm and 90 = 78.6 nm, Feret diameter: 41 ± 8 nm).

1) FHD: 5 μL of FuGENE HD (FHD, Roche, Mannheim, Germany) were added to 2 μg of pIRES-hrGFPII-*eIL-12 *or pIRES-hrGFPII-*rHMGB1 *at a total volume of 100 μL ddH_2_O, incubated for 10 minutes at room temperature and added to the seeded cells.

2) Plano-AuNP (EM CGC20, 20 nm; Plano GmbH, Wetzlar, Germany): 20 μL of Plano-AuNP were incubated for 24 h at room temperature with 2 μg of pIRES-hrGFPII-*eIL-12 *or pIRES-hrGFPII-*rHMGB1 *at a total volume of 95 μL ddH_2_O. For transfection 5 μL aliquots of FHD reagent (Roche, Mannheim, Germany) were added to 95 μL of the AuNP /vector suspension, incubated for 10 minutes at room temperature and added to cell cultures.

3) PLAL-AuNP size 1 (d50 = 28.5 nm and d90 = 43.4 nm hydrodynamic sizes; 14 ± 3 nm Feret diameter (Figure 6)) and size 2 (d50 = 52.4 nm and d90 = 78.6 nm hydrodynamic sizes; 41 ± 8 nm Feret diameter (Figure 6)): The PLAL-AuNP suspensions were sterilized by filtration through a 0.2 μm filter device (Millex-GV Sterilizing Filter Unit, Millipore, Billerica, USA). Subsequently, 20 μL of each sized AuNPs were incubated for 24 h at room temperature with 2 μg of pIRES-hrGFPII-*eIL-12 *or pIRES-hrGFPII-*rHMGB1 *at a total volume of 95 μL of ddH_2_O. For transfection 5 μL aliquots of FHD reagent (Roche, Mannheim, Germany) were added to 95 μL of the AuNP /vector suspension, incubated for 10 minutes at room temperature and added to cell cultures.

3.1) Nanoparticle generation: AuNPs were generated by pulsed laser ablation in liquid (PLAL) [9]. The beam of a femtosecond laser system (Spitfire Pro, Spectra-Physics), delivering 120 fs laser pulses at a wavelength of 800 nm was focused with a 40 mm lens on a 99.99% pure gold target placed at the bottom of a Petri dish filled with 2 mL of ddH_2_O. Pulse energy of 200 μJ at 5 kHz repetition rate was employed for 12 minutes of irradiation. The target position was set 4 mm or 2 mm below the determined focal point in air, in order to obtain colloidal suspensions containing AuNPs with mean hydrodynamic diameters of d_h _= 29 nm (size 1) and d_h _= 52 nm (size 2), respectively. The remaining small particles were removed by centrifugation. Characterisation of NP colloids was performed by dynamic light scattering using a Malvern Zetasizer ZS and by UV-Vis spectroscopy using a Shimadzu 1650.

4) Magnet-assisted transfection: (MA Lipofection & MATra-A):

4.1) MA Lipofection: 5 μL of FHD (Roche, Mannheim, Germany) were added to 2 μg of pIRES-hrGFPII-*eIL-12 *or pIRES-hrGFPII-*rHMGB1 *to a total volume of 97 μL ddH_2_O and incubated for 10 minutes at room temperature. Afterwards, 3 μL of MA Lipofection enhancer (PromoKine, Heidelberg, Germany) were added and incubated at room temperature for 15 minutes.

4.2) MATra-A: 3 μL of the magnetic reagent MATra-A (PromoKine, Heidelberg, Germany) were added to 2 μg of pIRES-hrGFPII-*eIL-12 *or pIRES-hrGFPII-*rHMGB1 *to a total volume of 97 μL of complete medium 199 (without FCS) and incubated for 15 minutes at room temperature.

For MATra-A and MA Lipofection, after final incubation, the 100 μL suspension was added to the cell cultures and each of the 6-well plates were placed on a magnetic plate at 37°C and 5% CO_2 _for 15 minutes (Universal Magnet Plate; PromoKine, Heidelberg, Germany). Afterwards, the plate was removed.

After each transfection, cells were incubated for 24 hours in complete medium 199 at 37°C and 5% CO_2_.

For each protocol the incubation of cells with the transfection reagents and without DNA was considered as the negative control.

The plasmid DNA uptake of pIRES-hrGFPII-*eIL-12 *and pIRES-hrGFPII-*rHMGB1 *was verified by fluorescence microscopy and measured by flow cytometry (FACSCalibur flow cytometer).

Each protocol was performed in triplicate.

Results are expressed as means.

### Transfection Efficiency Analyses

#### Fluorescence Microscopy

Transfected cells were fixed in a 4% paraformaldehyde/PBS solution for 15 minutes at room temperature. After fixation 10 μL of Vectashield Mounting Medium with DAPI (4'-6-diamidino-2-phenylindol, Vector Laboratories, Burlingame, CA, USA) was applied for fluorescent visualization of nucleic DNA. Fluorescence microscopy was performed using an Axio Imager. Z1 fluorescence microscope (Carl Zeiss MicroImaging GmbH, Jena, Germany) and images were recorded using the AxioVision Software (Rel. 4.7). The hrGFP fluorescence was measured employing wavelength filter set 10 (Carl Zeiss MicroImaging, Goettingen, Germany), while DAPI fluorescence was measured employing wavelength filter set 2.

### Flow cytometry

GFP expression of the transfected cells was analysed measuring green fluorescence by flow cytometry in order to determine the transfection efficiency of each protocol. Cells were trypsinized for 3-5 min, washed with PBS, resuspended in the medium, and measured with a FACScan flow cytometer (Becton, Dickinson and Company, Heidelberg, Germany). Fluorescence intensities were analysed with Cell Quest software (Becton, Dickinson and Company, Heidelberg, Germany). The percentage of positive cells was assessed comparing dot plot analysis of the transfected cells to cells incubated only with transfection reagent with or without the addition of NPs (depending of the protocol used).

Results are expressed as percentage of positive cells, as indicator for transfection efficiency.

The transfection efficiency results of each protocol were finally compared with those of the conventional FHD protocol.

### Toxicity Analyses

#### Flow cytometry

Propidium iodide (PI) staining was used to identify the cell death percentage after transfection. Cells were trypsinized, resuspended in complete medium 199 and PI (5 μg/mL) was added. The cytometry analysis was performed using a FACSCalibur device (Becton, Dickinson and Company, Heidelberg, Germany) with Cell Quest software (Becton, Dickinson and Company, Heidelberg, Germany). Thereafter, the cells were assessed for PI florescence by dot plot analysis and compared to cells incubated only with transfection reagent with or without the addition of NPs (depending of the protocol used).

### Results are expressed as percentage of positive cells

The toxicity results of each protocol were compared with those of the conventional FHD protocol.

### Proliferation Assay

Proliferation of cells in response to each transfection protocol was evaluated using a colorimetric cell proliferation ELISA (Roche Applied Science, Mannheim, Germany) which measures the incorporation of 5-bromo-2-deoxyuridine (BrdU), a thymidine analogue, into DNA by ELISA using an anti-BrdU monoclonal antibody.

Eight hours prior to transfection, 1.5 × 10^4 ^MTH53A cells were placed in 96-well plates. Cells were grown at 37°C and 5% CO_2 _in complete medium 199 (Invitrogen, Karlsruhe, Germany) supplemented with 10% heat-inactivated FCS (PAA Laboratories GmbH, Pasching, Austria), 200 U/ml penicillin and 200 ng/ml streptomycin (Biochrom AG, Berlin, Germany). Each protocol was performed in triplicate as explained above. The proliferation assay was carried out according to manufacturer's recommendations (Cell proliferation ELISA, colorimetric, Cat. No. 11647229001, Roche Applied Science, Mannheim, Germany). The reaction products were quantified by measuring the absorbance at 370 nm (reference wavelength 492 nm) using a scanning multiwell spectrophotometer equipped with the analysis software Gen 5 (Synergy HT multi-mode microplate reader, BioTek Instruments Inc., Bad Friedrichshall Germany). The absorbance results directly correlate to the amount of DNA synthesis and hereby to the number of proliferating cells.

Results are expressed as mean absorbance values

The proliferation results of each protocol were compared to those of non-transfected cells.

### Protein Expression

To confirm biological functionality of the expressed proteins, immunofluorescence directed against eIL-12 and canine HMGB1 was performed after transfection.

#### Equine IL-12

The expression of eIL-12 was evaluated in MTH53A cells. Eight hours prior to transfection 3 × 10^5 ^MTH53A cells were seeded in 6-well plates. Cells were grown under standard conditions as described above. Transfection was performed as explained for the FHD protocol. Subsequently, 24 h after transfection cells were fixed in a 4% paraformaldehyde/PBS solution for 20 minutes at room temperature, permeabilized and blocked. Immunofluorescence was performed using a goat IgG anti-p35 polyclonal primary antibody (IL-12 p35, sc-1280, Santa Cruz Biotechnology, Inc.; Santa Cruz, CA, USA; dilution 1:40) and a donkey anti-goat secondary antibody (IgG-TR, sc-2783; Santa Cruz Biotechnology, Inc.; Santa Cruz, CA; dilution 1:180).

Fluorescence microscopy was carried out using a Leica DMI 6000 fluorescence microscope (Leica Microsystems GmbH, Wetzlar Germany).

#### Canine HMGB1

The expression of HMGB1 was also evaluated in MTH53A cells. Cells were prepared as described for the eIL-12 expression. Twenty four hours after transfection with four different protocols (FHD, Plano-AuNP, PLAL-AuNP_S2 and Ma Lipofection), immunofluorescence was performed using an anti-HMGB1 mouse monoclonal antibody (HMGB1 antibody [HAP46.5], ab12029-100, Abcam, Cambridge, UK; 1:60) and a goat anti-mouse antibody (DyLight™ 549-TFP ester, Code Nr. 115-505-062, Jackson ImmunoResearch, West Grove, PA, USA; dilution 1:220). Fluorescence microscopy was also performed as described above.

### Statistics

Results are presented as mean ± standard deviation. Statistical significance was determined using the 1-tailed Wilcoxon-Mann-Whitney test. Differences were considered statistically significant for p ≤ 0.05.

## Competing interests

The authors declare that they have no competing interests.

## Authors' contributions

MCD carried out the construction of the expression vectors, the cell culture and DNA preparation, the transfections, the fluorescence and immunofluorescence microscopy analysis, the statistical analysis and the partial drafting of the manuscript. SW participated in the expression vector design and construction, in cell culture, fluorescence and immunofluorescence microscopy analysis and partial drafting of the manuscript. AB carried out the PLAL-AuNPs generation. AM assisted MCD in performing flow cytometry analysis. JM participated in the IL 12 vector design. JTS took part in the expression vector design and construction. SB performed the supervision of the PLAL-AuNPs generation. IN and KF, head of the Small Animal Clinic and the Equine Clinic, participated in the conception design of the study. HME carried out the principal study design, partial drafting and finalisation of the manuscript and the supervision of the molecular and cell biological work. All authors read and approved the final manuscript.

## References

[B1] Baumgart J Bintig W Ngezahayo A Willenbrock S Murua Escobar H Ertmer W Lubatschowski H Heisterkamp A Quantified femtosecond laser based opto-perforation of living GFSHR-17 and MTH53 a cells Opt Express 2008 16 3021 3031 10.1364/OE.16.003021 18542388

[B2] Petersen S Soller JT Wagner S Richter A Bullerdiek J Nolte I Barcikowski S Murua Escobar H Co-transfection of plasmid DNA and laser-generated gold nanoparticles does not disturb the bioactivity of GFP-HMGB1 fusion protein J Nanobiotechnology 2009 7 6 10.1186/1477-3155-7-6 19852831 PMC2775017

[B3] Bertram J MATra - Magnet Assisted Transfection: combining nanotechnology and magnetic forces to improve intracellular delivery of nucleic acids Curr Pharm Biotechnol 2006 7 277 285 10.2174/138920106777950825 16918404

[B4] Ghosh P Han G De M Kim CK Rotello VM Gold nanoparticles in delivery applications Adv Drug Deliv Rev 2008 60 1307 1315 10.1016/j.addr.2008.03.016 18555555

[B5] Rosi NL Giljohann DA Thaxton CS Lytton-Jean AK Han MS Mirkin CA Oligonucleotide-modified gold nanoparticles for intracellular gene regulation Science 2006 312 1027 1030 10.1126/science.1125559 16709779

[B6] Borm PJ Robbins D Haubold S Kuhlbusch T Fissan H Donaldson K Schins R Stone V Kreyling W Lademann J Krutmann J Warheit D Oberdorster E The potential risks of nanomaterials: a review carried out for ECETOC Part Fibre Toxicol 2006 3 11 10.1186/1743-8977-3-11 16907977 PMC1584248

[B7] Dahl JA Maddux BL Hutchison JE Toward greener nanosynthesis Chem Rev 2007 107 2228 2269 10.1021/cr050943k 17564480

[B8] Sylvestre J Poulin S Kabashin AV Sacher E Meunier M Luong JHT Surface Chemistry of Gold Nanoparticles Produced by Laser Ablation in Aqueous Media J Phys Chem B 2004 108 16864 16869 10.1021/jp047134+

[B9] Petersen S Barcikowski S In Situ Bioconjugation: Single Step Approach to Tailored Nanoparticle-Bioconjugates by Ultrashort Pulsed Laser Ablation Adv Funct Mater 2009 19 1167 1172 10.1002/adfm.200801526

[B10] Luo D Saltzman WM Enhancement of transfection by physical concentration of DNA at the cell surface Nat Biotechnol 2000 18 893 895 10.1038/78523 10932162

[B11] Kamau SW Hassa PO Steitz B Petri-Fink A Hofmann H Hofmann-Amtenbrink M von Rechenberg B Hottiger MO Enhancement of the efficiency of non-viral gene delivery by application of pulsed magnetic field Nucleic Acids Res 2006 34 e40 10.1093/nar/gkl035 16540591 PMC1408310

[B12] Schakowski F Gorschluter M Junghans C Schroff M Buttgereit P Ziske C Schottker B Konig-Merediz SA Sauerbruch T Wittig B Schmidt-Wolf IG A novel minimal-size vector (MIDGE) improves transgene expression in colon carcinoma cells and avoids transfection of undesired DNA Mol Ther 2001 3 793 800 10.1006/mthe.2001.0322 11356084

[B13] Chithrani BD Ghazani AA Chan WC Determining the size and shape dependence of gold nanoparticle uptake into mammalian cells Nano Lett 2006 6 662 668 10.1021/nl052396o 16608261

[B14] Yin W Xiang P Li Q Investigations of the effect of DNA size in transient transfection assay using dual luciferase system Anal Biochem 2005 346 289 294 10.1016/j.ab.2005.08.029 16213455

[B15] Renker B Diestel S Ruonala M Bertram J MATra ein Trojanisches Pferd fuer eine zellschonende Transfektion BIOspektrum 2010 1 2

[B16] Pan Y Neuss S Leifert A Fischler M Wen F Simon U Schmid G Brandau W Jahnen-Dechent W Size-dependent cytotoxicity of gold nanoparticles Small 2007 3 1941 1949 10.1002/smll.200700378 17963284

[B17] Sperling RA Rivera Gil P Zhang F Zanella M Parak WJ Biological applications of gold nanoparticles Chem Soc Rev 2008 37 1896 1908 10.1039/b712170a 18762838

[B18] Connor EE Mwamuka J Gole A Murphy CJ Wyatt MD Gold nanoparticles are taken up by human cells but do not cause acute cytotoxicity Small 2005 1 325 327 10.1002/smll.200400093 17193451

[B19] Taylor U Klein S Petersen S Kues W Barcikowski S Rath D Nonendosomal cellular uptake of ligand-free, positively charged gold nanoparticles Cytometry A 77 439 446 10.1002/cyto.a.20846 20104575

[B20] Shukla R Bansal V Chaudhary M Basu A Bhonde RR Sastry M Biocompatibility of gold nanoparticles and their endocytotic fate inside the cellular compartment: a microscopic overview Langmuir 2005 21 10644 10654 10.1021/la0513712 16262332

[B21] Pernodet N Fang X Sun Y Bakhtina A Ramakrishnan A Sokolov J Ulman A Rafailovich M Adverse effects of citrate/gold nanoparticles on human dermal fibroblasts Small 2006 2 766 773 10.1002/smll.200500492 17193121

[B22] Brandenberger C Rothen-Rutishauser B Muhlfeld C Schmid O Ferron GA Maier KL Gehr P Lenz AG Effects and uptake of gold nanoparticles deposited at the air-liquid interface of a human epithelial airway model Toxicol Appl Pharmacol 242 56 65 10.1016/j.taap.2009.09.014 19796648

